# A hybrid CNN–BiLSTM network with additive attention for automated four-stage classification of Alzheimer's disease from brain MRI

**DOI:** 10.3389/frai.2026.1948601

**Published:** 2026-10-16

**Authors:** T. Deenadayalan, S. P. Shantharajah, Swarna Priya Ramu

**Affiliations:** School of Computer Science Engineering and Information Systems, Vellore Institute of Technology (VIT), Vellore, India

**Keywords:** Alzheimer's disease, attention mechanism, bidirectional LSTM, class imbalance, convolutional neural network, deep learning, explainable AI, magnetic resonance imaging

## Abstract

AD is a progressive and irreversible neurodegenerative disorder. Because the structural changes that accompany the earliest stages of the disease are subtle and easily overlooked, manual interpretation of MRI is time-consuming and prone to inter-observer disagreement. This study presents an end-to-end deep learning framework that fuses spatial and sequential reasoning to classify brain MRI into four clinically meaningful stages: Person with no dementia, Person with very mild dementia, Person with mild dementia, and Person with moderate dementia. An additive attention mechanism subsequently weights the most informative positions before a compact dense head produces the final decision. Class imbalance is addressed by capped oversampling of real minority slices combined with residual cost-sensitive weighting, applied to the training fold only; pixel-space SMOTE is deliberately avoided because interpolating between two unregistered MRI slices produces images without an anatomical referent. Exact-duplicate screening is applied prior to partitioning, and a quantitative near-duplicate audit is reported for the fixed folds. Trained on 6,400 grayscale MRI slices under a stratified 70/15/15 protocol, the proposed CNN–BiLSTM–Attention model attains a test accuracy of 95.94%, a balanced accuracy of 96.08%, an MCC of 0.9334, a Cohen's kappa of 0.9325 and a macro-averaged one-vs.-rest AUC of 0.9954, on a slice-level held-out test fold. Per-class analysis shows strong sensitivity and specificity for the three better-represented stages; the extremely rare Person with moderate dementia class (nine test images) shows a favorable point estimate but is too small to support a claim of robust detection on its own. Attention maps and Grad-CAM overlays localize the evidence the network uses, and an error-structure analysis shows that no misclassification spans more than two stages. Because the corpus carries no subject identifiers, patient-grouped partitioning is not possible and the reported accuracies should be interpreted as an upper bound attainable under a slice-level split; this constraint is quantified explicitly and motivates the external-validation study outlined in the manuscript. Overall, the results highlight the methodological value of coupling convolutional feature extraction with bidirectional sequence modeling and additive attention under a leakage-aware, imbalance-aware evaluation protocol for multi-stage AD staging.

## 1 Introduction

Dementia is an umbrella term for a family of syndromes characterized by a decline in memory, reasoning, language, and the capacity to carry out everyday activities. Among its many forms, AD is the most common and is a leading cause of disability and dependency in older adults. The disorder has an insidious onset: neuropathological changes may begin years or even decades before patients or their families observe the earliest symptoms. By the time overt cognitive impairment is apparent, substantial and largely irreversible neuronal loss has usually already occurred. This prolonged preclinical interval makes accurate stage assignment important not only for diagnosis, but also for research applications such as cohort stratification and longitudinal follow-up, in which subtle structural differences must be detected reliably.

In clinical workflows, sMRI is widely used because it is non-invasive, broadly available, and sensitive to micro- and macro-structural changes—such as ventricular enlargement and hippocampal or medial temporal atrophy—that track disease progression (Jack et al., 2008; Chandra et al., 2019). Translating these subtle morphometric changes into consistent stage labels nevertheless remains difficult: stage boundaries are not sharply defined, and scanner- and site-level differences introduce nuisance variation. Inter-reader disagreement is also common, particularly for very mild or borderline presentations. These challenges make AD staging a valuable testbed for methodological advances in medical image classification, particularly approaches that (i) extract discriminative structure from subtle differences and (ii) evaluate models rigorously under class imbalance and potential leakage.

Deep learning has reshaped medical image analysis over the past decade by learning task-specific representations directly from data (LeCun et al., 2015; Litjens et al., 2017). Convolutional neural networks capture local texture and shape cues and are widely used as backbones for image-based diagnosis (Li et al., 2022). Nevertheless, AD staging is not purely a local-pattern problem: clinically relevant cues are spatially distributed and often expressed as gradual structural changes across neighboring regions. Recurrent architectures such as long short-term memory (LSTM) networks can model ordered dependencies (Hochreiter and Schmidhuber, 1997), and bidirectional variants incorporate context from both directions (Schuster and Paliwal, 1997). Attention mechanisms further enable models to focus computation on informative spatial locations rather than aggregating evidence uniformly (Guo et al., 2022; Han et al., 2023). A hybrid CNN–BiLSTM–Attention design is therefore well motivated for slice-wise MRI staging: CNNs extract rich spatial descriptors, sequence modeling captures contextual continuity across the feature map, and attention highlights discriminative regions.

A second challenge concerns data quality and evaluation rigor. Public AD MRI datasets are often highly imbalanced—the Person with moderate dementia class may contain only a few scans against thousands for Person with no dementia—which can bias a classifier toward majority classes and reduce sensitivity to rare but clinically important advanced cases; near-duplicate images can also cross train/validation/test boundaries, inflating reported performance and undermining reproducibility (Yagis et al., 2021; Wen et al., 2020). Robust pipelines should therefore prioritize de-duplication, stratified partitioning and training-only balancing, and report imbalance-aware metrics such as balanced accuracy and MCC alongside accuracy.

Motivated by these observations, this work proposes and evaluates a hybrid CNN–BiLSTM–Attention network for four-class Alzheimer's staging (Person with no dementia, Person with very mild dementia, Person with mild dementia, and Person with moderate dementia). The principal contributions are:

1. We design a hybrid architecture in which a deep convolutional backbone is serialized into 64 spatial tokens—one per cell of the final 8 × 8 convolutional grid—which are then processed by a bidirectional LSTM equipped with an additive attention block. Preserving the full two-dimensional grid, rather than collapsing the column axis into eight row-aggregated vectors, is what allows the learned attention distribution to be folded back into an interpretable spatial map.
2. We adopt a leakage-aware protocol: exact duplicates are removed before splitting, the stratified partition is performed before any balancing, and all class-imbalance handling is confined to the training fold. We additionally report a quantitative near-duplicate audit computed on the fixed folds, and state explicitly the limits it places on the interpretation of absolute accuracy.
3. We replace pixel-space SMOTE with capped oversampling of *real* minority slices combined with residual cost-sensitive weighting (Cui et al., 2019), on the grounds that linear interpolation between two unregistered MRI slices in raw pixel space produces images with no anatomical referent.
4. We report a comprehensive evaluation—balanced accuracy, Matthews correlation coefficient, Cohen's kappa, per-class and macro-averaged AUC, per-class metrics, and bootstrap confidence intervals—together with an analysis of the clinical severity of the errors the model makes.
5. We provide model-level interpretability through both the network's own additive attention distribution and gradient-based Grad-CAM (Selvaraju et al., 2017), so that the anatomical evidence supporting each decision can be inspected.

## 2 Literature survey

Automated AD analysis has progressed from hand-crafted morphometric descriptors toward end-to-end deep learning pipelines. Early deep-learning studies demonstrated that CNNs can extract discriminative biomarkers directly from sMRI slices or volumes; for example, convolutional models for AD classification from T1-weighted MRI have been reported in high-impact journals (Bae et al., 2020), and 3D CNNs have been applied to volumetric neuroimaging to exploit full spatial context (Payan and Montana, 2018). Compared with 2D slice-based methods, volumetric approaches can exploit full 3D context but often require heavier computation and more carefully standardized preprocessing.

The reproducible benchmark of Wen et al. (2020) is particularly relevant to the present study. Evaluating 2D-slice, 2D-patch, 3D-patch, ROI-based and 3D-subject CNN variants under a common, subject-level protocol on ADNI and AIBL, that work reports that (i) subject-level 3D approaches are generally the strongest configuration, and (ii) a substantial fraction of the accuracies previously published for 2D slice-level pipelines are attributable to data leakage rather than to the architecture. Yagis et al. (2021) isolate the same effect experimentally, showing that slice-level partitioning of brain MRI can inflate reported accuracy by tens of percentage points relative to subject-level partitioning of the identical data. Both findings motivate the leakage audit reported in Section 3.3 and the framing of our absolute accuracies as upper bounds under a slice-level split.

Beyond single-modality sMRI, multimodal fusion has been explored to integrate complementary disease signals: Lu et al. (2018) combined structural MR with FDG-PET using a multiscale deep network, illustrating how metabolic and anatomical cues can be fused when such data are available, though multimodal acquisition is not always feasible in routine screening, reinforcing the value of strong sMRI-only models. The medical-imaging community continues to emphasize recurring practical hurdles such as limited labeled data, domain shift, and severe class imbalance (Litjens et al., 2017).

A particularly influential stream targets multi-stage dementia staging. Murugan et al. (2021) proposed DEMNET for four-class staging using SMOTE-based balancing; Afzal et al. (2019) investigated augmentation strategies for imbalance. Transfer learning has also been widely adopted: Sharma et al. (2022) combined transfer learning with classical machine learning, and transfer-learning-driven multiclass staging from MRI has been reported in Q1/Q2 journals Khan et al., (2023). Deenadayalan and Shantharajah (2022) reported improved early AD detection and diagnosis performance using deep learning. Ensembles have been used to improve robustness, as in (Loddo et al. 2022), and recent work explores lightweight, feature-selective variants to reduce computational cost and overfitting risk (Poloni and Ferrari, 2022; Mahendran and Vincent, 2022); classical analyses remain important reference points for benchmarking (Wolz et al., 2011).

Recent literature from 2024 to 2026 has increasingly focused on hybrid deep learning architectures, transformer-based models, and improved optimization strategies for MRI-based Alzheimer's disease analysis. Classical machine-learning and deep-learning approaches have also been systematically compared, highlighting the importance of learned representations in classification performance (Aslan and Özüpak, 2024). Transformer and hybrid convolution–transformer architectures have further been explored for Alzheimer's disease staging, including MaxViT-based classification (Aslan et al., 2025) and ResNet101 combined with a transformer encoder (Almalki et al., 2025). These approaches demonstrate the potential of combining local convolutional features with global feature interactions. The proposed model follows a related principle through lightweight additive attention applied to the spatial feature tokens, while retaining a recurrent BiLSTM component for sequential feature modeling.

Recent four-class staging studies have also investigated self-attention with ResNet architectures and Bayesian optimization (Yaqoob et al., 2024), as well as fine-tuned CNN-based transfer learning approaches (Hussain et al., 2025). Other recent studies have explored optimization strategies for deep-learning models (Aslan, 2024) and reinforcement-learning-based approaches for continuous control (Aslan et al., 2023). Collectively, these developments highlight the growing importance of hybrid representation learning and robust optimization in medical-image analysis, providing the broader methodological context for the CNN–BiLSTM–Attention framework proposed in this study.

Hybrid architectures that graft sequence models onto convolutional backbones (CNN–LSTM and attention-augmented variants) attempt to capture structured spatial dependencies that a flat global-pooling classifier can underuse. These developments are built upon standard deep-learning components: LSTM (Hochreiter and Schmidhuber, 1997), bidirectional recurrence (Schuster and Paliwal, 1997), attention mechanisms (Guo et al., 2022), and regularization/optimization practices such as normalization (Chang et al., 2020), adaptive optimization (Sun and Zhao, 2020), cosine learning-rate scheduling with warm restarts (Loshchilov and Hutter, 2017), and label smoothing (Szegedy et al., 2016). In parallel, generative approaches including GANs (Wang et al., 2021) have been explored for data enrichment, and explainable AI methods for medical image analysis have been surveyed extensively (van der Velden et al., 2022); Deenadayalan et al. (2026) and Shantharajah et al. (2025) have also reported related explainability-orientated medical-imaging studies.

Deenadayalan and Shantharajah (2026) proposed PredatorNet, a transfer-learning-driven pipeline for multi-stage Alzheimer's classification from brain MRI, illustrating continued interest in attention mechanisms and efficient model design for staging tasks. Complementary prognostic modeling directions have also been explored; for example, an AI-based prognostic survival analysis framework has been reported for AD diagnosis and progression modeling using MRI data (Deenadayalan and Shantharajah, 2025).

Table 1 summarizes representative studies and highlights common methodological choices. Two columns have been added in this revision—partition granularity and explicit leakage control—because these, rather than architecture, are the dimensions along which reported accuracies are least comparable.

**Table 1:** Representative deep-learning studies on Alzheimer's disease classification and staging from neuroimaging.

References	Modality and method	Cls.	Imbalance strategy	Partition granularity	Leakage control reported
Bae et al. (2020)	sMRI; CNN-based AD classification	2	Not stated	Slice/subject	Not stated
Payan and Montana (2018)	MRI; 3D CNN on volumes	2–3	Not stated	Subject (volumetric)	Implicit (volume-level)
Li et al. (2019)	MRI; hierarchical fully convolutional network (3D)	2–3	Not stated	Subject (volumetric)	Implicit (volume-level)
Lu et al. (2018)	sMRI + FDG-PET; multiscale fusion network	2–3	Not stated	Subject	Not stated
Wen et al. (2020)	MRI; reproducible benchmark of 2D/3D CNN variants	2–3	Not applicable	Subject	Explicit; quantifies leakage
Yagis et al. (2021)	MRI; controlled study of slice- vs. subject-level splitting	2	Not applicable	Both, compared	Explicit; quantifies leakage
Afzal et al. (2019)	MRI; augmentation-focused pipeline	4	Augmentation	Slice	Not stated
Khan et al. (2023)	MRI; transfer learning for staging	4	Not stated	Slice	Not stated
Murugan et al. (2021)	MRI; DEMNET (CNN)	4	SMOTE	Slice	Not stated
Sharma et al. (2022)	MRI; transfer learning + ML classifier	4	Not stated	Slice	Not stated
Loddo et al. (2022)	MRI; deep ensemble	4	Not stated	Slice	Not stated
Poloni and Ferrari (2022); Mahendran and Vincent (2022)	MRI; lightweight/feature-selective models	4	Varies	Slice	Not stated
Aslan et al. ( (2025)	MRI; MaxViT hybrid transformer	4	Not stated	Slice	Not stated
Almalki et al. (2025)	MRI; ResNet101 + transformer encoder	4	Not stated	Slice	Not stated
Deenadayalan and Shantharajah (2022)	MRI; deep learning for earlier detection/diagnosis	n/s	Not stated	Not stated	Not stated
This work	MRI; CNN–BiLSTM–Attention, capped real-image oversampling	4	Capped oversampling + residual weights	Slice	Explicit; audit reported

The final two columns, added in revision, record the partition granularity and the leakage controls each study reports; “not stated” means the information could not be determined from the published description.

## 3 Materials and methods

### 3.1 Dataset description and provenance

Experiments were conducted on the publicly available, preprocessed *Alzheimer's Disease* brain-MRI collection distributed through Kaggle, with four classes: Person with no dementia (Non_Demented), Person with very mild dementia (Very_Mild_Demented), Person with mild dementia (Mild_Demented), and Person with moderate dementia (Moderate_Demented). The same corpus has been used in DEMNET (Murugan et al., 2021) and several subsequent four-stage staging studies (Sharma et al., 2022; Loddo et al., 2022; Yaqoob et al., 2024; Hussain et al., 2025). The dataset contains 6,400 single-channel axial MRI slices distributed across the four classes, as summarized in Table 2.

**Table 2:** Dataset composition by class after exact-duplicate screening.

Class	Label index	Image count	Proportion
Person with no dementia	0	3,200	50.00%
Person with very mild dementia	1	2,240	35.00%
Person with mild dementia	2	896	14.00%
Person with moderate dementia	3	64	1.00%
Total	–	6,400	100%

The dataset is distributed in preprocessed form and does not provide complete acquisition or subject-level information. In particular, field strength, sequence parameters, voxel spacing, acquisition site, and subject identifiers are unavailable. The upstream preprocessing procedure is also not fully documented. Consequently, protocol homogeneity and subject-level grouping cannot be independently assessed.

The preprocessing applied in this study consisted of grayscale decoding, bilinear resizing to 128 × 128, and intensity rescaling to [0, 1]. No additional registration, bias-field correction, or intensity harmonization was performed. Slice-wise learning was adopted to match the available data format and provide a computationally efficient formulation for the proposed model.

The class distribution is highly imbalanced, with 3,200 persons with no dementia, 2,240 persons with very mild dementia, 896 persons with mild dementia, and only 64 persons with moderate dementia images (Figure 1). This imbalance can bias classification toward the majority classes; therefore, explicit imbalance handling was applied during training as described in Section 3.4.

**Figure 1:**
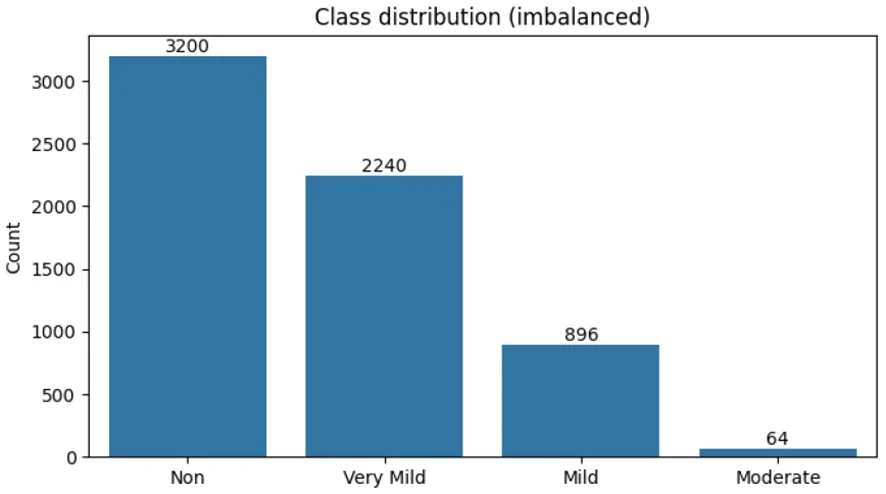
Class distribution of the dataset before balancing. The Person with moderate dementia class contains only 64 images compared with 3,200 images in the Person with no dementia class.

Representative slices from the four disease stages are shown in Figure 2.

**Figure 2:**
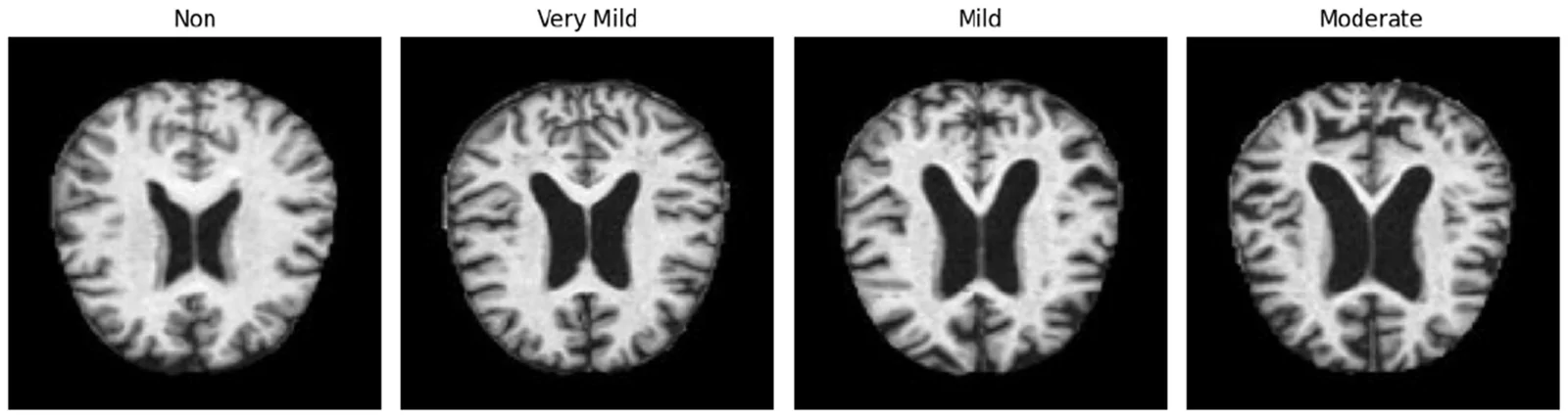
Representative axial MRI slices for the four disease stages.

Before dataset partitioning, an exact-duplicate screen was performed using MD5 hashing of the resized images. No byte-identical duplicates were identified. This screening reduces the possibility of exact duplicate images occurring across different folds. However, exact-duplicate screening does not address highly similar but non-identical slices; therefore, a separate near-duplicate leakage audit is presented in Section 3.3.

### 3.2 Data partitioning

Data were divided with a stratified 70/15/15 split into training, validation, and test folds. The 70% training fraction provides sufficient data for fitting the deep model, the 15% validation fold supports model selection, and the 15% test fold provides an untouched estimate of generalization. The resulting split counts are summarized in Table 3. Fold disjointness was asserted programmatically at the index level, and the split was performed *before* any balancing, so that no resampled, reweighted or augmented instance can reach the validation or test folds.

**Table 3:** Class distribution across the stratified 70/15/15 split and the capped-oversampled training fold used for fitting.

Class	Train	Val	Test	Train (after capped oversampling)
Person with no dementia	2,240	480	480	2,240
Person with very mild dementia	1,568	336	336	2,240
Person with mild dementia	627	134	135	2,240
Person with moderate dementia	45	10	9	450
Total	4,480	960	960	7,170

Validation and test folds are identical to the original submission and are never modified.

### 3.3 Near-duplicate leakage audit and the limits of slice-level splitting

The redistributed dataset does not contain subject identifiers, scan identifiers, or filename information from which participant identity can be recovered. Consequently, patient-level grouped partitioning cannot be performed reliably on this dataset. The evaluation was therefore conducted using slice-level partitioning, and the resulting performance should not be interpreted as an estimate of subject-level generalization.

To quantify the potential overlap between the training and test folds, a near-duplicate audit was performed, with the resulting distribution of maximum cosine similarity shown in Figure 3. Each test slice was downscaled to 32 × 32, *L*_2_-normalized, and compared with all training slices using cosine similarity. For each test slice, the maximum similarity to any training slice was retained. As shown in Table 4, no byte-level duplicate was identified at a cosine similarity threshold of 0.999. However, 874 of 960 test slices (91.0%) had a training counterpart with similarity ≥0.99, while 938 (97.7%) and all 960 test slices (100%) had a counterpart at thresholds of ≥0.98 and ≥0.95, respectively.

**Figure 3:**
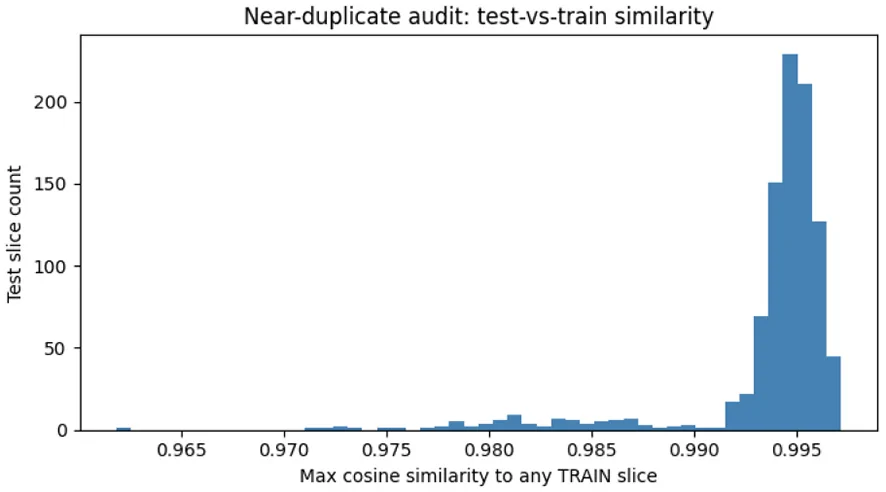
Near-duplicate audit showing the distribution of maximum cosine similarity between each test slice and the training fold. The high concentration of similarities above 0.99 indicates substantial similarity between held-out test slices and training samples under the slice-level partitioning protocol.

**Table 4:** Near-duplicate audit of the fixed test fold against the fixed training fold.

Cosine-similarity threshold	Test slices with a training match	Percentage
≥0.999	0/960	0.0%
≥0.990	874/960	91.0%
≥0.980	938/960	97.7%
≥0.950	960/960	100.0%

For each of the 960 test slices, the maximum cosine similarity to any of the 4,480 training slices was computed using 32 × 32 *L*_2_-normalized representations.

This high similarity is consistent with the characteristics of slice-level partitioning, where adjacent axial slices or closely related augmented samples can occur across different folds. Therefore, the internal performance values should be interpreted as an upper-bound estimate under the available slice-level protocol rather than as evidence of patient-level generalization.

The leakage affects the absolute performance estimates but does not change the fact that all architectural and imbalance configurations were evaluated using the same fixed folds. Consequently, the ablation results provide consistent within-protocol comparisons, although they should not be interpreted as unbiased estimates of performance on unseen patients. Similarly, comparisons with previous studies using the same corpus should be interpreted within the context of their respective partitioning strategies.

The external OASIS evaluation described in Section 5.11 provides an additional assessment using an independent data source. Nevertheless, definitive subject-level generalization requires datasets with reliable subject identifiers and subject-disjoint evaluation. This remains an important direction for future validation.

### 3.4 Near-duplicate leakage audit and the limits of slice-level splitting

The redistributed dataset does not contain subject identifiers, scan identifiers, or filename information from which participant identity can be recovered. Consequently, patient-level grouped partitioning cannot be performed reliably on this dataset. The evaluation was therefore conducted using slice-level partitioning, and the resulting performance should not be interpreted as an estimate of subject-level generalization.

To quantify the potential overlap between the training and test folds, a near-duplicate audit was performed. Each test slice was downscaled to 32 × 32, *L*_2_-normalized, and compared with all training slices using cosine similarity. For each test slice, the maximum similarity to any training slice was retained. As shown in Table 4, no byte-level duplicate was identified at a cosine similarity threshold of 0.999. However, 874 of 960 test slices (91.0%) had a training counterpart with similarity ≥0.99, while 938 (97.7%) and all 960 test slices (100%) had a counterpart at thresholds of ≥0.98 and ≥0.95, respectively.

This high similarity is consistent with the characteristics of slice-level partitioning, where adjacent axial slices or closely related augmented samples can occur across different folds. Therefore, the internal performance values should be interpreted as an upper-bound estimate under the available slice-level protocol rather than as evidence of patient-level generalization.

The leakage affects the absolute performance estimates but does not change the fact that all architectural and imbalance configurations were evaluated using the same fixed folds. Consequently, the ablation results provide consistent within-protocol comparisons, although they should not be interpreted as unbiased estimates of performance on unseen patients. Similarly, comparisons with previous studies using the same corpus should be interpreted within the context of their respective partitioning strategies.

The external OASIS evaluation described in Section **??** provides an additional assessment using an independent data source. Nevertheless, definitive subject-level generalization requires datasets with reliable subject identifiers and subject-disjoint evaluation. This remains an important direction for future validation.

### 3.5 Class imbalance: from pixel-space SMOTE to capped real-image oversampling

The original training procedure used SMOTE (Chawla et al., 2002) to balance the training classes. However, pixel-space interpolation is not well suited to MRI slices because interpolating two flattened images can generate synthetic patterns that do not necessarily correspond to anatomically plausible brain structures. This limitation is particularly relevant when the images are not voxel-aligned through subject-level registration. Therefore, the final training strategy uses real MRI slices rather than pixel-space synthetic images.

Class imbalance was addressed using two complementary mechanisms applied only to the training fold. First, minority classes were oversampled by repeating real images toward the majority-class count, with a maximum duplication factor of 10 × . This produced a training set of 7,170 real images with class counts of [2240, 2240, 2240, 450], corresponding to duplication factors of 1.00, 1.43, 3.57, and 10.00, respectively. On-the-fly augmentation was applied during training so that repeated samples were subjected to stochastic transformations rather than being presented as identical images.

Second, residual class imbalance was addressed using tempered cost-sensitive weighting. The class weights were calculated as:


$$w_{c}={\left(\frac{\max_{j}n_{j}}{n_{c}}\right)}^{\gamma },\;\gamma =0.5,$$

resulting in weights ranging from 0.765 to 1.706. This approach provides additional emphasis to the minority class without allowing its contribution to dominate the optimization process.

The capped real-image oversampling strategy avoids the generation of potentially unrealistic pixel-space interpolations while maintaining sufficient representation of minority classes during training. Pixel-space SMOTE was retained only as a comparison condition in the imbalance ablation study (Section 5.9), allowing its effect to be evaluated experimentally rather than assumed.

A conservative training-only augmentation policy was used, consisting of horizontal flipping, rotation of ±0.05 turns, zoom of up to 8%, and translation of up to 5%. These transformations were selected to preserve the anatomical structure of the MRI slices. Augmentation was disabled during validation and testing and was not applied during inference.

### 3.6 Model configuration and hyperparameters

The network was implemented using TensorFlow/Keras 2.20 and optimized with Adam (Sun and Zhao, 2020) using label-smoothed categorical cross-entropy (Szegedy et al., 2016). A deterministic learning-rate schedule was employed, consisting of a five-epoch linear warmup to 10^−3^ followed by cosine decay to 10^−5^ (Loshchilov and Hutter, 2017). Model selection was based on validation macro-averaged *F*_1_, which was used for checkpointing and early stopping. Gradient-norm clipping with a clipnorm of 1.0 was also applied to improve training stability.

The main architecture and training hyperparameters are summarized in Table 5.

**Table 5:** Training configuration and hyperparameters of the proposed CNN–BiLSTM–Attention model.

Parameter	Value
Input resolution	128 × 128 × 1 (grayscale)
Convolutional blocks	4 blocks; 2 convolutional layers per block (32/64/128/256 filters)
Feature-to-sequence transform	Reshape (8, 8, 256) → (64, 256), producing 64 spatial tokens
Recurrent layer	1 × bidirectional LSTM (128 units per direction), return_sequences=True
Attention	Additive (Bahdanau-style) attention over 64 tokens; Dense(64, tanh) → Dense(1) → softmax
Dense head	128 units (ReLU, L2 = 10^−4^) → BatchNorm → 4-way softmax
Dropout	0.3 after recurrent block; 0.4 in dense head
Optimizer	Adam; peak learning rate = 10^−3^; gradient clipnorm = 1.0
Loss	Categorical cross-entropy; label smoothing = 0.05
Imbalance strategy	Capped oversampling (cap 10 × ) + residual weights (γ = 0.5)
Batch size / Maximum epochs	64 / 100
Learning-rate schedule	5-epoch linear warmup to 10^−3^, followed by cosine decay to 10^−5^
Model selection	Validation macro-*F*_1_ for checkpointing and early stopping; patience = 30
Trainable parameters	1,619,237
Random seed	42

### 3.7 Reproducibility considerations

To support reproducibility, all experiments were conducted under a fixed random seed (Table 5). The leakage-aware order of operations was strictly enforced: (i) resizing and hashing for de-duplication, (ii) stratified splitting into train/validation/test folds, (iii) the near-duplicate audit computed on the fixed folds, and (iv) training-only balancing and training-only augmentation. The validation fold was used for model selection only; the test fold was not consulted at any point during model development. Hyperparameters and training controls are reported in full to facilitate independent replication.

### 3.8 Computational considerations and the 2D–3D trade-off

The proposed model adopts a two-dimensional slice-wise formulation, which involves a trade-off between computational efficiency and preservation of volumetric anatomical information. This consideration is particularly relevant for Alzheimer's disease, where structural characteristics such as hippocampal and medial temporal atrophy and ventricular enlargement are inherently three-dimensional.

A 2D slice provides only a planar representation of these structures. Consequently, properties such as volumetric atrophy must be inferred from individual cross-sections, making the prediction potentially sensitive to slice position. A volumetric 3D model can instead exploit spatial relationships across multiple anatomical planes and may therefore provide more complete structural information.

The 2D formulation nevertheless provides important computational advantages. It requires substantially less memory than full-volume 3D convolutional networks, permits larger effective batch sizes, and is compatible with the available redistributed dataset, which contains individual MRI slices rather than complete volumes or subject identifiers. Reconstructing artificial volumes from these slices would not provide a reliable basis for a meaningful 3D comparison.

Previous comparative work has reported advantages for 3D and ROI-based approaches over conventional 2D slice-based models when evaluated using subject-level protocols (Wen et al., 2020). These findings, together with the leakage identified in Section 3.3, indicate that the current 2D results should be interpreted as slice-level performance and should not be taken as evidence that slice-wise modeling is superior to volumetric approaches.

A direct 3D baseline is therefore not included in the present experiment. A meaningful 3D comparison requires complete volumetric data, consistent 3D preprocessing, and subject-level partitioning. Such evaluation is more appropriately performed using independent cohorts that provide volumetric MRI data and subject identifiers. Accordingly, future work will investigate 3D and hybrid 2D–3D architectures under subject-disjoint evaluation to determine whether volumetric context provides additional benefits for disease-stage classification.

### 3.9 Evaluation metrics

Performance was assessed using complementary metrics appropriate for multi-class staging under class imbalance (Powers, 2011; Chicco and Jurman, 2020). Overall accuracy is computed as (Equation 1) (Powers, 2011):

$$\begin{array}{l} \text{Acc}=\frac{\sum_{k=1}^{K}{\text{TP}}_{k}}{N} \end{array}$$ (1)

where *N* is the total number of test samples and TP_*k*_ denotes true positives for class *k*. To reduce majority-class dominance, balanced accuracy (mean recall across classes) is reported (Equation 2) (Powers, 2011; Chicco and Jurman, 2020):


$$\begin{array}{l} \text{BalAcc}=\frac{1}{K}\overset{K}{\underset{k=1}{\sum }}\frac{{\text{TP}}_{k}}{{\text{TP}}_{k}+{\text{FN}}_{k}}. \end{array}$$ (2)

Per-class precision and recall (sensitivity) are computed in a one-vs.-rest manner as shown in Equation 3, while specificity and F1-score are defined in Equation 4 (Powers, 2011; Chicco and Jurman, 2020):


Preck=TPkTPk+FPk,Reck=TPkTPk+FNk (3)

$$\begin{array}{ll} \text{Spe}{\text{c}}_{k}=\frac{\text{T}{\text{N}}_{k}}{\text{T}{\text{N}}_{k}+\text{F}{\text{P}}_{k}}, & F{\text{l}}_{k}=\frac{2{\mathrm{Prec}}_{k}{\mathrm{Rec}}_{k}}{{\mathrm{Prec}}_{k}+{\mathrm{Rec}}_{k}} \end{array}$$ (4)

To quantify chance-corrected agreement, Cohen's kappa is reported (Equation 5) (Cohen, 1960; Chicco and Jurman, 2020):


$$\begin{array}{l} \kappa =\frac{p_{o}-p_{e}}{1-p_{e}}, \end{array}$$ (5)

where *p*_*o*_ is the observed agreement and *p*_*e*_ is the expected agreement under independence. The Matthews correlation coefficient, a correlation-based summary that remains informative under severe imbalance, is reported in its multi-class form (Equation 6) (Chicco and Jurman, 2020):


$$\begin{array}{l} \text{MCC}=\frac{N\sum_{k}{\text{TP}}_{k}-\sum_{k}p_{k}t_{k}}{\sqrt{\left(N^{2}-\sum_{k}p_{k}^{2}\right)\left(N^{2}-\sum_{k}t_{k}^{2}\right)}}, \end{array}$$ (6)

where *t*_*k*_ is the number of samples whose true class is *k* and *p*_*k*_ the number predicted as *k*.

Threshold-independent behavior is analyzed using one-vs.-rest ROC curves (Fawcett, 2006) and precision–recall (PR) curves; PR analysis is particularly informative under class imbalance (Saito and Rehmsmeier, 2015).

Three statistical instruments have been added in this revision.

- **Areas under the curve**. Per-class one-vs.-rest AUC (Fawcett, 2006) and average precision are computed and tabulated, so separability is a number rather than a visual inference from a curve.
- **Bootstrap confidence intervals**. Percentile bootstrap 95% CIs over 2,000 resamples are reported for accuracy, balanced accuracy, macro-*F*_1_ and MCC (Efron and Tibshirani, 1993), essential given that the person with moderate dementia test support is nine images: one flipped prediction moves recall by 0.111, so a point estimate alone would mislead.
- **Paired significance testing**. Ablation arms are compared against the baseline with the exact McNemar test on paired predictions over the same 960 test slices (McNemar, 1947), so architectural claims are statistical statements rather than two point estimates.

## 4 Proposed methodology

The system is an end-to-end trainable network that performs feature extraction, sequential reasoning, and attention-based aggregation within a single graph. It proceeds in five stages: (i) a convolutional backbone converts an input slice into a compact feature tensor; (ii) the tensor is serialized into an ordered sequence of spatial tokens; (iii) bidirectional recurrence models contextual dependencies across the sequence; (iv) additive attention aggregates the most informative tokens into a fixed-length context vector; and (v) a dense head outputs four class probabilities. The overall architecture and the surrounding protocol are shown in Figure 4.

**Figure 4:**
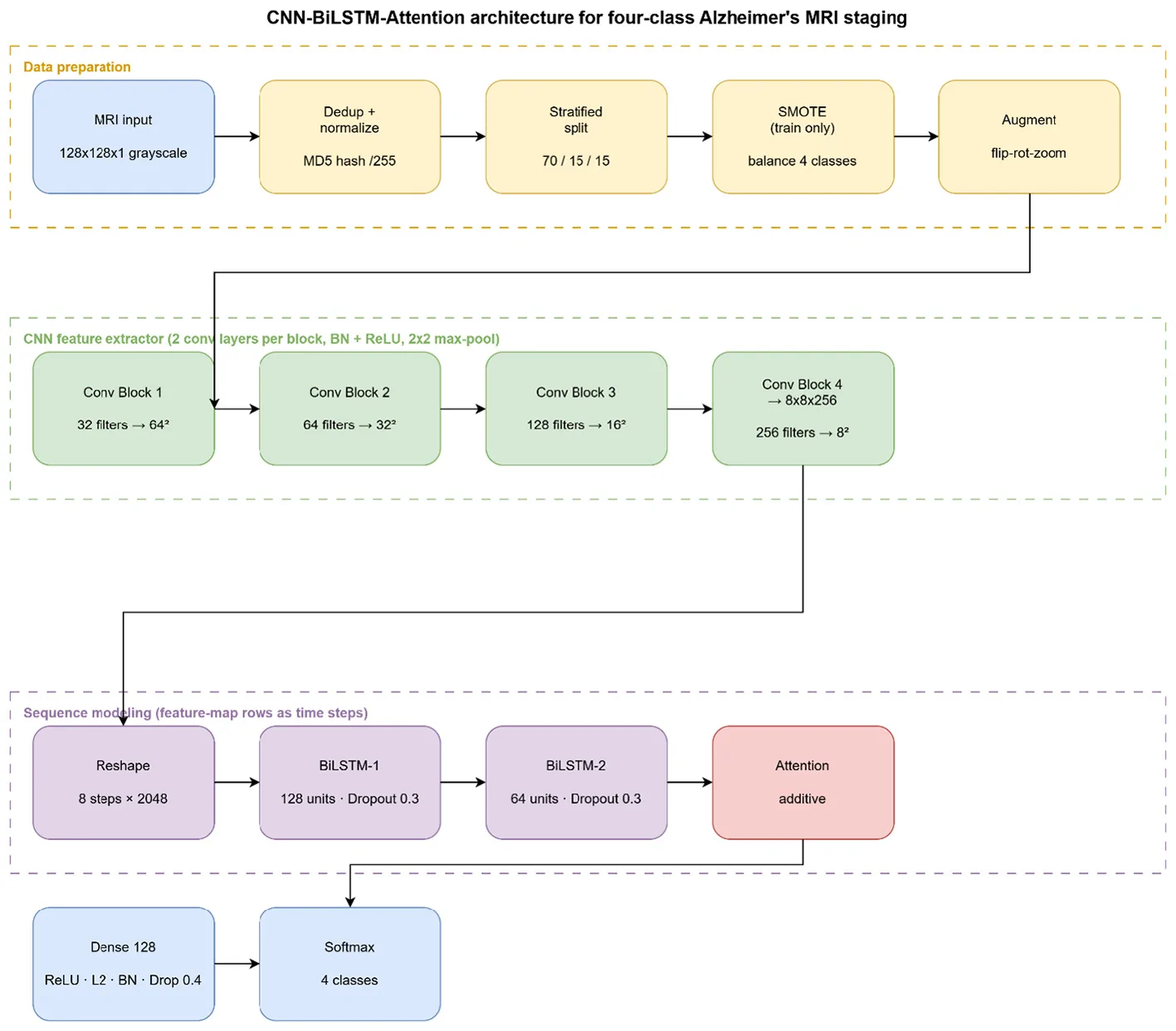
Overall architecture of the proposed CNN–BiLSTM–Attention model, redrawn for this revision. Band A shows the leakage-aware data preparation, including the near-duplicate audit and the replacement of SMOTE by capped real-image oversampling; band B shows the network, in which the 8 × 8 × 256 tensor is serialized into 64 spatial tokens rather than eight row-vectors; band C shows the evaluation and interpretability outputs computed on the untouched test fold.

### 4.1 Convolutional feature extractor

The backbone comprises four blocks, each applying two 3 × 3 convolutions with batch normalization (Chang et al., 2020) and ReLU, followed by 2 × 2 max pooling. The filter count doubles across blocks (32, 64, 128, 256), yielding an 8 × 8 × 256 tensor from a 128 × 128 × 1 input.

The design follows a local-to-global progression: early layers respond to edges and fine texture, while later layers encode higher-level morphology that is more stable with respect to small intensity fluctuations and noise. Batch normalization stabilizes training, and pooling progressively increases the receptive field while controlling parameter count.

### 4.2 Spatial tokenization and sequence modeling

Let **F** ∈ ℝ^8 × 8 × 256^ be the final convolutional tensor. It is serialized in row-major order into a sequence of *T* = 64 tokens (Equation 7):


$$\begin{array}{l} {\left\{{\text{x}}_{t}\right\}}_{t=1}^{64},\;{\text{x}}_{t}\in {\mathbb{R}}^{256},\;{\text{x}}_{t}=\text{F}\left[\left\lfloor t/8\right\rfloor ,t\;\mathrm{mod}\;8,:\right]. \end{array}$$ (7)

Each token is thus the 256-channel descriptor of one cell of the 8 × 8 grid, corresponding to a 16 × 16-pixel region of the input.

This differs from the original submission, which reshaped the same tensor to (8, 8·256), giving eight row-aggregated tokens of dimension 2,048. Three consequences motivate the change:

1. **The column axis is preserved**. Under the row-aggregated form the eight patch descriptors of a row are concatenated before the recurrent layer sees them, so the model can relate rows but not left-to-right within a row—the wrong axis for a task dominated by bilateral medial structures.
2. **The sequence is long enough for recurrence to matter**. With eight timesteps a BiLSTM is effectively an expensive MLP; with 64 timesteps of 256 features a *single* BiLSTM layer suffices, shrinking the recurrent block from ~2.39M to 0.39M parameters (1,619,237 total).
3. **Attention becomes spatially interpretable**. Token *t* maps to grid cell (⌊*t*/8⌋, *t* mod 8), so the 64 attention weights fold back into an 8 × 8 map overlayable on the input slice (Section 5.7); the row-aggregated form gave only an eight-element vector that could not localize horizontally.

A single bidirectional LSTM (Hochreiter and Schmidhuber, 1997; Schuster and Paliwal, 1997) with 128 units per direction processes the sequence with return_sequences enabled, producing 64 contextualized hidden states ${\left\{{\text{h}}_{t}\right\}}_{t=1}^{64}$ of dimension 256, followed by dropout (Srivastava et al., 2014).

Although MRI is not a temporal signal, this sequential reinterpretation models spatial continuity and long-range interactions across the feature map without a full 3D network; bidirectionality incorporates context from both scan directions of the serialized grid.

### 4.3 Additive attention and classification head

Given the hidden states ${\left\{h_{t}\right\}}_{t=1}^{64}$, additive attention (Guo et al., 2022) first computes an attention score *e*_*t*_ and normalized attention weight α_*t*_ for each token, as defined in Equation 8. The resulting attention-weighted context vector is then obtained according to Equation 9:


$$\begin{array}{l} e_{t}={\text{v}}^{⊤}\tanh\left(\text{W}{\text{h}}_{t}+\text{b}\right),\;\alpha_{t}=\frac{\exp\left(e_{t}\right)}{\sum_{j=1}^{64}\exp\left(e_{j}\right)}, \end{array}$$ (8)

$$\begin{array}{l} \text{c}=\overset{64}{\underset{t=1}{\sum }}\alpha_{t}{\text{h}}_{t}, \end{array}$$ (9)

where **c** ∈ ℝ^256^ is the context vector. The scoring path is implemented as Dense(64, tanh) followed by a linear Dense(1) projection and a softmax over the token axis. In the original implementation the tanh nonlinearity was applied directly to the scalar score immediately before the softmax, which bounds the ratio between the largest and smallest attention weight at *e*^2^≈7.4 and forces a near-uniform distribution; moving the nonlinearity into the hidden projection removes that constraint. A dense layer (128 ReLU units with L2 weight decay), batch normalization and dropout map **c** to a four-way softmax.

### 4.4 Training algorithm

Algorithm 1 illustrates the proposed leakage-aware training and evaluation workflow, which provides its procedural representation.

Algorithm 1 **Leakage-aware training and evaluation workflow for the CNN–BiLSTM–Attention model**

 **Input:** MRI image folders; image size *S* = 128 
 **Output:** Trained model *M* and test-set performance report
1:  Read each image as grayscale; resize to *S*×*S*; compute MD5 hash; discard exact duplicates.
2:  Normalize pixels to [0, 1]; reshape to (*S, S*, 1).
3:  Perform a stratified split into train/validation/test folds (70/15/15); assert fold disjointness.
4:  Audit near-duplicate exposure of the fixed test fold against the fixed training fold; report it.
5:  Balance the training fold only: oversample real minority slices with a 10 × cap, then apply residual cost-sensitive sample weights.
6:  Construct *M*: convolutional blocks → reshape to 64 spatial tokens → single BiLSTM → additive attention → dense head → softmax.
7:  Train with Adam (Sun and Zhao, 2020) and label-smoothed cross-entropy (Szegedy et al., 2016), using training-only augmentation, a warmup-plus-cosine learning-rate schedule (Loshchilov and Hutter, 2017), and macro-*F*_1_-based checkpointing and early stopping.
8:  Evaluate on the untouched test fold; report all metrics with bootstrap confidence intervals, and compute attention and Grad-CAM explanations.

## 5 Results and discussion

### 5.1 Training behavior

Training ran for the full 100 epochs under the deterministic warmup-plus-cosine schedule; early stopping on validation macro-*F*_1_ did not trigger, and the best checkpoint was restored at the end. Figure 5 shows accuracy, loss, the two selection metrics, and the realized learning rate. The learning-rate panel is a regression test on the optimization fix: it shows a smooth warmup-and-cosine curve rather than the staircase of plateau-triggered reductions that characterized the earlier schedule.

Validation accuracy and loss are visibly noisier than their training counterparts during the first sixty epochs. This is expected and is a direct consequence of the fold composition rather than of instability in the model: the validation fold contains ten Person with moderate dementia images, so a handful of flipped predictions on that class produces a large excursion in macro-averaged quantities. Both selection metrics converge and flatten over the final twenty epochs, when the cosine tail has reduced the learning rate below 10^−4^.

### 5.2 Overall performance

The corresponding one-vs.-rest ROC and precision–recall curves for the four disease stages are shown in Figure 6. Balanced accuracy and MCC are particularly informative under class imbalance because they reduce the dominance of the majority class and quantify agreement between predictions and labels even when class supports are skewed. Headline metrics with 95% percentile bootstrap confidence intervals are given in Table 6.

**Figure 5:**
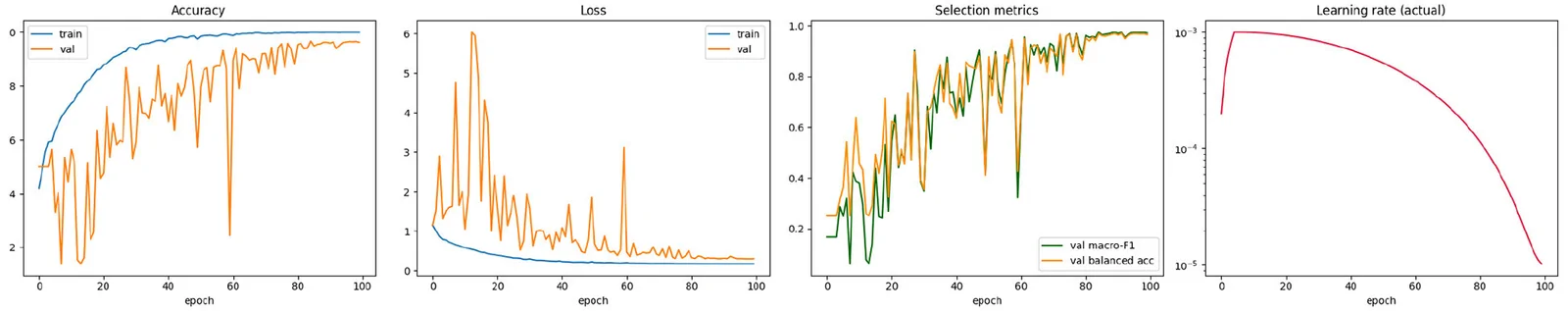
Training dynamics. From left: training and validation accuracy; training and validation loss; the two validation selection metrics (macro-*F*_1_ and balanced accuracy); and the realized learning rate on a logarithmic scale, showing the five-epoch linear warmup followed by cosine decay.

**Table 6:** Test-set performance of the proposed model.

Metric	Value	95% bootstrap CI
Accuracy	0.9594	[0.9469, 0.9708]
Balanced accuracy	0.9608	[0.9480, 0.9733]
Matthews correlation coefficient (MCC)	0.9334	[0.9130, 0.9523]
Cohen's kappa	0.9325	–
Macro-averaged F1	0.9677	[0.9568, 0.9777]
Weighted-averaged F1	0.9592	–
Macro-averaged one-vs.-rest AUC	0.9954	–

95% percentile bootstrap confidence intervals over 2,000 resamples of the test fold are reported where applicable.

The confidence intervals are narrow for the aggregate metrics—accuracy [0.9469, 0.9708] over 2,000 resamples—which is what the 960-image test fold supports. They are not, and cannot be, narrow for the Person with moderate dementia class considered alone; that class is discussed separately in Section 3.8.

### 5.3 Validation-vs.-test gap and the question of overfitting

The validation and test performance of the proposed model were compared to assess the consistency of its performance across the two held-out folds. As shown in Table 7, the validation accuracy was 0.9667, compared with 0.9594 on the test set, giving a difference of 0.0073. Similarly, the differences were 0.0078 for balanced accuracy, 0.0064 for macro-*F*_1_, and 0.0117 for MCC. Cohen's κ differed by 0.0123, whereas macro-AUC was slightly higher on the test set, with a difference of −0.0006.

**Table 7:** Validation-vs.-test performance comparison for the proposed model.

Metric	Validation	Test	Validation −Test
Accuracy	0.9667	0.9594	+0.0073
Balanced accuracy	0.9686	0.9608	+0.0078
MCC	0.9452	0.9334	+0.0117
Cohen's κ	0.9448	0.9325	+0.0123
Macro-*F*_1_	0.9741	0.9677	+0.0064
Macro-AUC	0.9948	0.9954	−0.0006

The relatively small validation–test differences indicate consistent performance across the two folds and do not, by themselves, provide strong evidence of conventional overfitting. The validation fold was used for checkpoint selection and early stopping, whereas the test fold was evaluated after training. The close agreement between the two folds therefore suggests that the selected model maintained similar performance on unseen test slices.

However, this result should be interpreted within the limitations of the slice-level evaluation protocol. The near-duplicate audit reported in Section 3.3 identified substantial similarity between training and held-out slices. Consequently, the small validation–test gap should be interpreted as evidence of consistency between the two held-out folds, rather than as evidence of subject-level generalization or absence of dataset leakage.

Overall, the validation–test comparison indicates a small performance gap under the current experimental protocol, while the external OASIS evaluation in Section 5.11 provides a more stringent assessment of generalization under cross-dataset domain shift.

### 5.4 Threshold-independent behavior and areas under the curve

To assess threshold-independent classification behavior, one-vs.-rest ROC and precision–recall curves were evaluated for the four disease stages. The corresponding AUC and average precision values are reported in Table 8, while the curves are shown in Figure 7. All four classes achieved ROC-AUC values above 0.99, with a macro-AUC of 0.9954. The average precision values were also high across the classes, ranging from 0.972 for persons with mild dementia to 1.000 for persons with moderate dementia. These results indicate strong class separability on the internal test set. However, the very small support of the person with moderate dementia class should be considered when interpreting its perfect AUC and average precision.

**Table 8:** Per-class one-vs.-rest ROC-AUC and average precision on the test set.

Class	AUC	Average precision	Support
Person with no dementia	0.995	0.995	480
Person with very mild dementia	0.994	0.990	336
Person with mild dementia	0.992	0.972	135
Person with moderate dementia	1.000	1.000	9
Macro average	0.9954	–	960

**Figure 6:**
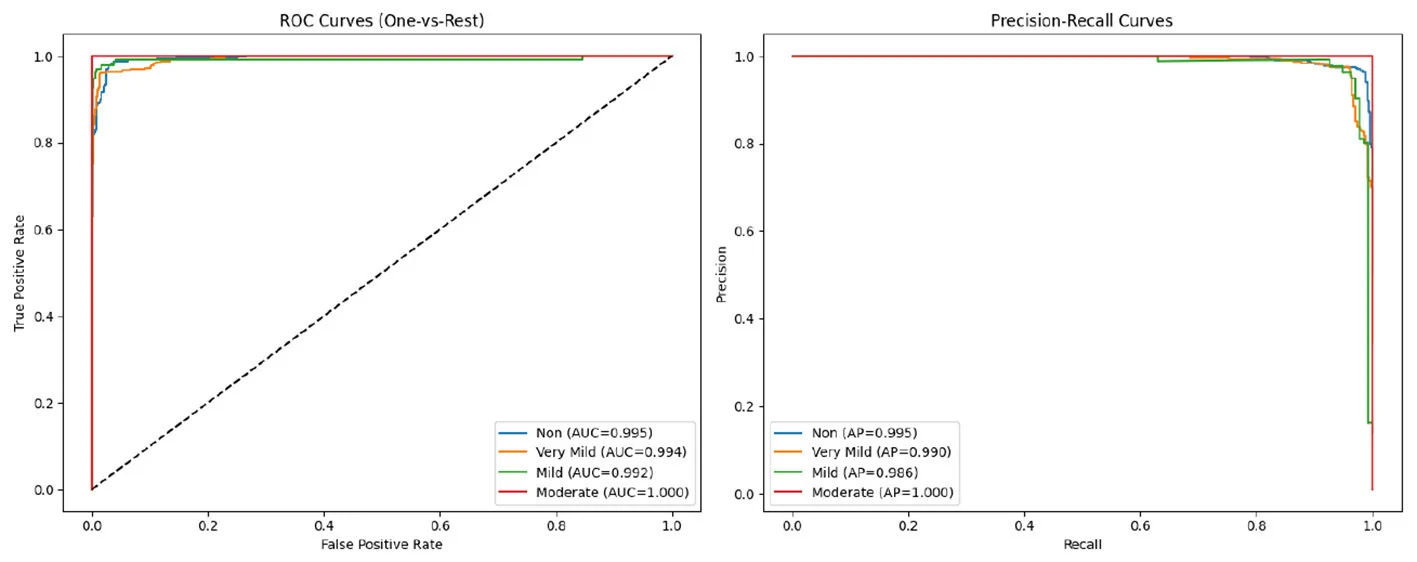
One-vs.-rest ROC and precision–recall curves for the four disease stages.

### 5.5 Clinical interpretation of the error structure

The 39 test-set misclassifications were analyzed according to ordinal distance between the true and predicted disease stages and according to the direction of the error (Table 9). This provides additional insight beyond overall accuracy by distinguishing adjacent-stage errors from larger staging deviations.

**Table 9:** Decomposition of the 39 test-set errors by ordinal distance and direction of staging error.

Error type	Count	%	Constituent confusions
Adjacent stage (|Δ| = 1)	32	82.1%	Very mild dementia → no dementia (23); no dementia → very mild dementia (4); Mild dementia → very mild dementia (3); very mild dementia → mild dementia (2)
Two stages (|Δ| = 2)	7	17.9%	Mild dementia → no dementia (7)
Three stages (|Δ| = 3)	0	0.0%	–
Under-staging	33	84.6%	Very mild dementia → no dementia (23); Mild dementia → no dementia (7); Mild dementia → very mild dementia (3)
Over-staging	6	15.4%	no dementia → very mild dementia (4); very mild dementia → mild dementia (2)
Total	39	100%	4.06% of 960 test slices

Assigning ordinal indices from 0 to 3 to the four stages, 32 of the 39 errors (82.1%) were adjacent-stage confusions, while seven errors (17.9%) spanned two stages. No error spanned three stages, and no confusion occurred directly between persons with no dementia and persons with moderate dementia. Thus, the majority of errors remained within one disease stage of the reference label.

The direction of the errors was asymmetric. A total of 33 errors (84.6%) represented under-staging, compared with six errors (15.4%) representing over-staging. The most frequent error was persons with very mild dementia being predicted as persons with no dementia, accounting for 23 errors. This pattern indicates that the earliest disease stage represents the principal source of classification error and may be particularly important when considering early-stage screening applications.

When the four classes are collapsed into a binary distinction between dementia and no dementia, the model achieved a sensitivity of 450/480 = 93.8% and a specificity of 476/480 = 99.2%. The 30 false negatives comprised 23 persons with very mild dementia and 7 persons with mild dementia cases. These results suggest strong binary discrimination on the evaluated test set, although the analysis remains conditional on the reference labels and the slice-level evaluation protocol.

Because the dataset does not provide inter-rater reliability information, some errors, particularly those occurring near the no dementia–very mild dementia boundary, may also reflect ambiguity in the reference labels. Therefore, the observed error structure should be interpreted as a characterization of model behavior on the available labels rather than as a definitive assessment of clinical staging performance.

### 5.6 Qualitative predictions

Figure 7 shows twelve randomly selected test slices with true label, predicted label and softmax confidence, allowing direct inspection of whether confidence behaves sensibly at the adjacent-stage boundaries where (Section 5.5) essentially all errors occur. Correct predictions dominate, and the misclassifications that appear are adjacent-stage confusions carrying visibly lower confidence than correct predictions.

### 5.7 Model interpretability: attention maps and Grad-CAM

#### 5.7.1 Attention maps

The additive attention distribution is a forward-pass quantity the network actually uses to decide, not a *post-hoc* reconstruction. Since token *t* corresponds to grid cell (⌊*t*/8⌋, *t* mod 8) of the final convolutional feature map, the 64 attention weights fold back into an 8 × 8 spatial map, each cell covering a 16 × 16-pixel receptive region (Figures 8, 9). The distribution is measurably selective rather than uniform: against a uniform entropy of log64 = 4.159 nats, the mean observed entropy across the four illustrated cases is 3.410 nats (no dementia 3.47, very mild 3.92, mild 3.04, and moderate 3.22). The two more advanced stages draw the most concentrated attention, consistent with focal, gross structural change, while very mild dementia attracts the most diffuse attention, consistent with distributed, subtle evidence; the class-mean maps concentrate on the periventricular and inferior-medial bands where ventricular enlargement and medial temporal atrophy are expressed.

**Figure 7:**
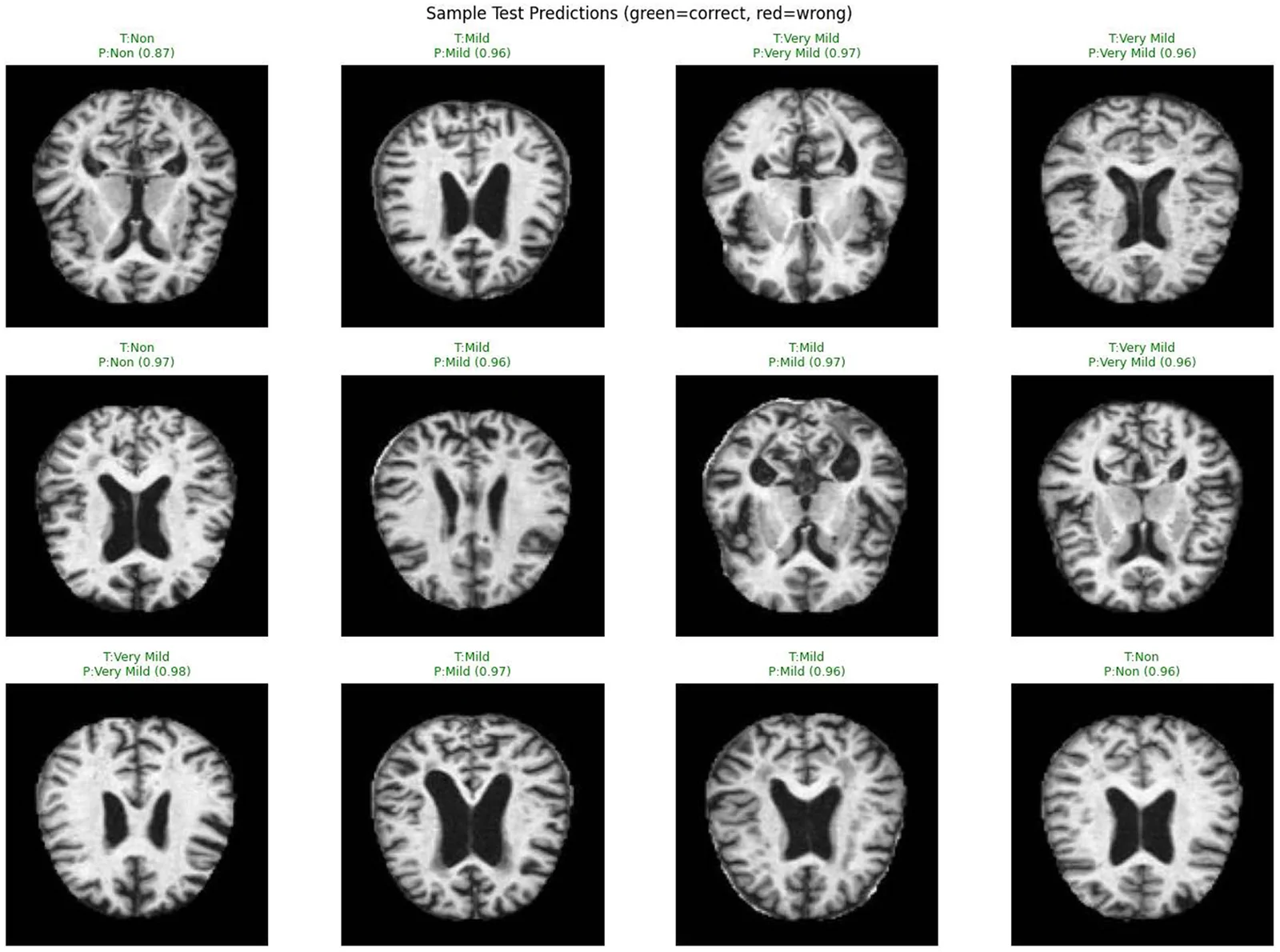
Sample test-set predictions. Titles show the true label (T) and predicted label (P) with softmax confidence; green indicates a correct prediction and red an incorrect one.

**Figure 8:**
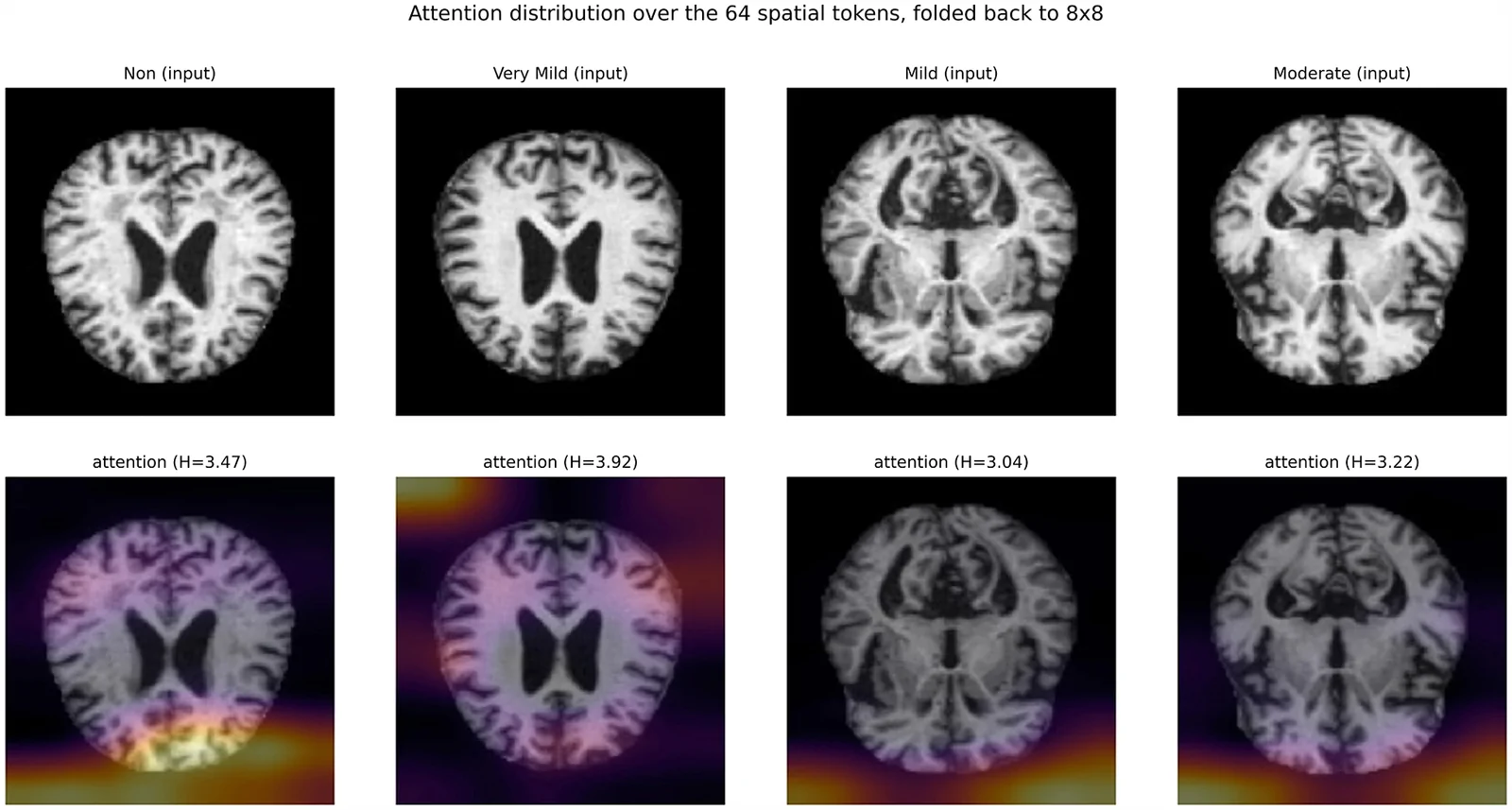
Additive-attention distributions over the 64 spatial tokens, folded back into an 8 × 8 map and overlaid on the input slice. Top row: one correctly classified test slice per stage. Bottom row: the corresponding attention overlay, annotated with the entropy *H* of the distribution in nats (uniform attention over 64 tokens would give *H* = 4.159).

#### 5.7.2 Grad-CAM

Grad-CAM (Selvaraju et al., 2017) is gradient-based and class-conditional, weighting the last convolutional layer's channels by the mean gradient of the predicted class score. Figure 10 shows overlays for the same four cases; the two methods agree on the broad localization, which is the useful check—attention shows where the model pooled evidence, Grad-CAM shows where the class score is sensitive to it.

**Figure 9:**
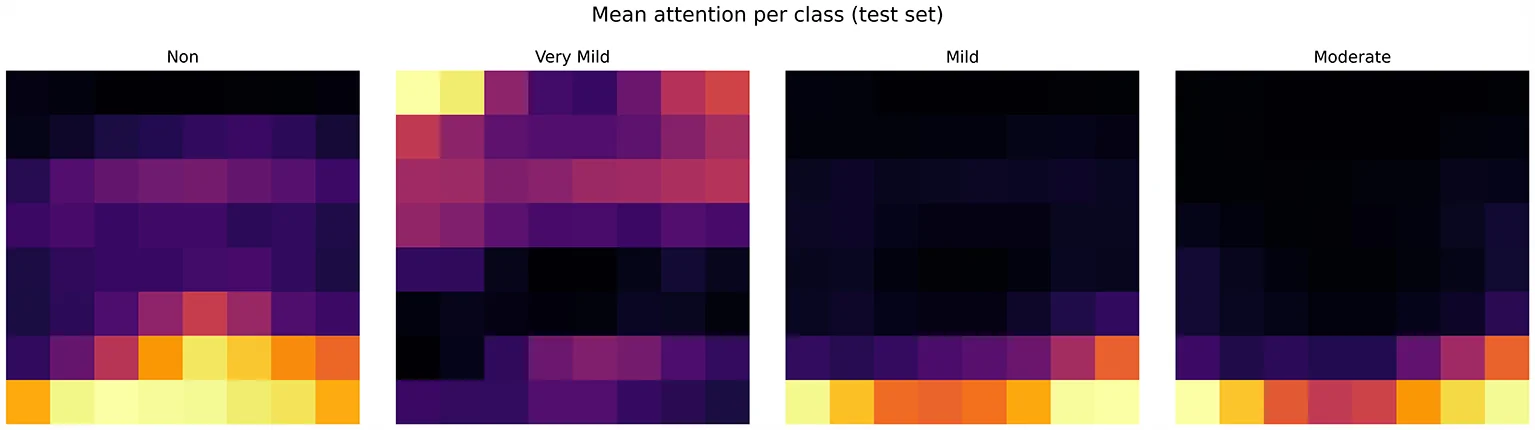
Mean attention map per class over the test fold, shown on the native 8 × 8 token grid. Brighter cells receive greater average attention.

**Figure 10:**
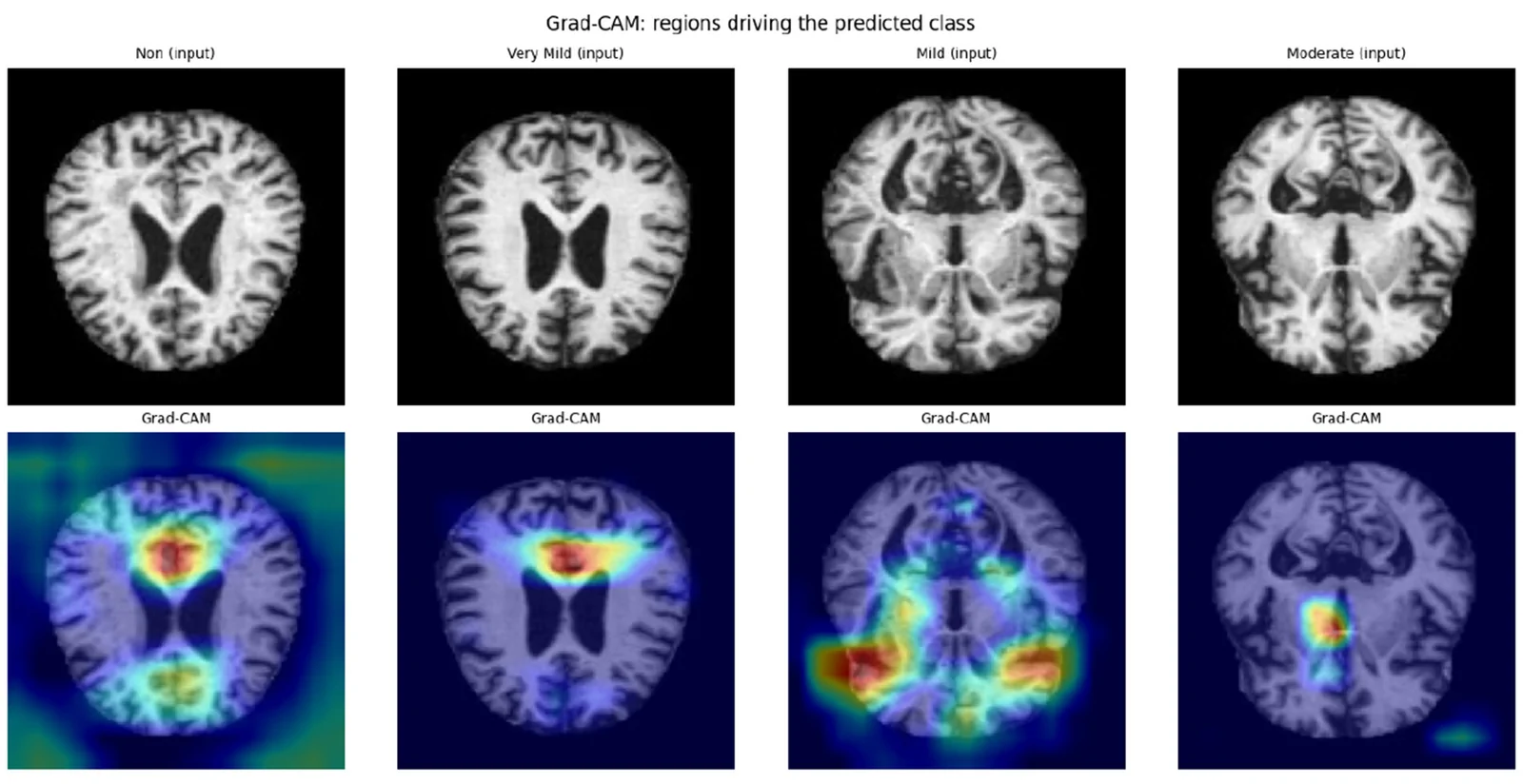
Grad-CAM overlays for the same four representative slices, computed on the final convolutional layer and conditioned on the predicted class.

#### 5.7.3 Scope

The maps have 16 × 16-pixel resolution and are regional, not anatomical: they indicate attention to periventricular structure but cannot segment a hippocampus or quantify its volume, and are not evidence of clinical correctness. A rigorous evaluation would overlay the maps on registered images with structural labels and compute agreement with expert-annotated regions of interest, which requires a cohort distributing that metadata (Section 5.11). We present the explanations as evidence of non-trivial, anatomically plausible selection, and claim nothing stronger.

### 5.8 Ablation study 1: contribution of the architectural components

An architectural ablation study was performed to investigate the individual and combined contributions of the major components of the proposed architecture. Four model configurations were evaluated: (i) CNN only, (ii) CNN + BiLSTM, (iii) CNN + Attention, and (iv) CNN + BiLSTM + Attention, where the latter represents the complete proposed architecture. The CNN-only configuration consists of the convolutional backbone followed by global average pooling over the 64 feature tokens. The CNN + BiLSTM configuration introduces bidirectional recurrent modeling followed by global average pooling over the resulting hidden representations, without the attention mechanism. The CNN + Attention configuration employs attention-based feature aggregation with a learnable positional embedding, without the recurrent component. The complete CNN + BiLSTM + Attention configuration integrates both bidirectional sequence modeling and attention-based feature aggregation.

All four configurations were evaluated on the same test set comprising 960 paired test slices using the same class-imbalance handling strategy. The parameter counts of the CNN-only, CNN + BiLSTM, CNN + Attention, and CNN + BiLSTM + Attention configurations were 1,208,484, 1,602,724, 1,241,381, and 1,619,237, respectively. The complete results are presented in Table 10.

Table 10Architectural ablation results on the test set.

**Table:** 

Architecture	Params	Accuracy	Bal. Acc.	MCC	κ	Macro-*F*_1_	Macro-AUC	Mod. recall
CNN only	1,208,484	0.6333	0.7756	0.4891	0.4606	0.6581	0.8949	1.0000
CNN + BiLSTM	1,602,724	0.6146	0.5587	0.4444	0.4137	0.5193	0.8807	0.2222
CNN + Attention	1,241,381	0.5188	0.6095	0.2766	0.2541	0.5907	0.8153	0.7778
CNN + BiLSTM + Attention	1,619,237	**0.9594**	**0.9608**	**0.9334**	**0.9325**	**0.9677**	**0.9954**	**1.0000**

**Table:** 

**Comparison**	**Exact McNemar** *p***-value**
CNN only vs. CNN + BiLSTM	0.2602
CNN only vs. CNN + Attention	8.616 × 10^−9^
CNN only vs. CNN + BiLSTM + Attention	2.128 × 10^−76^

All configurations were evaluated on the same 960 test slices. Bold values indicate the best performance among the compared architectural configurations; tied values, where applicable, are also shown in bold.

The CNN-only baseline achieved an accuracy of 0.6333, balanced accuracy of 0.7756, and macro-*F*_1_ score of 0.6581. Although the moderate-stage recall reached 1.0000, the lower overall accuracy and macro-*F*_1_ score indicate that the convolutional representation alone did not provide equally strong discrimination across all disease stages. The corresponding MCC, Cohen's κ, and macro-AUC values were 0.4891, 0.4606, and 0.8949, respectively.

The addition of the BiLSTM module without attention resulted in lower performance. The CNN + BiLSTM configuration obtained an accuracy of 0.6146, balanced accuracy of 0.5587, and macro-*F*_1_ score of 0.5193. Its moderate-stage recall decreased to 0.2222, while MCC and Cohen's κ were 0.4444 and 0.4137, respectively. The macro-AUC was 0.8807. The exact McNemar test, performed using paired predictions from the same 960 test slices, yielded *p* = 0.2602 when compared with the CNN-only baseline (McNemar, 1947). This indicates that the difference in paired classification outcomes between the CNN-only and CNN + BiLSTM configurations was not statistically significant.

The CNN + Attention configuration achieved an accuracy of 0.5188, balanced accuracy of 0.6095, and macro-*F*_1_ score of 0.5907. Moderate-stage recall was 0.7778, while MCC and Cohen's κ were 0.2766 and 0.2541, respectively. The macro-AUC was 0.8153. The exact McNemar test against the CNN-only baseline produced *p* = 8.616 × 10^−9^, indicating a statistically significant difference in paired predictions(McNemar, 1947). However, the lower accuracy, balanced accuracy, macro-*F*_1_, MCC, and macro-AUC demonstrate that attention-based aggregation alone did not provide sufficient discriminative performance for the evaluated task.

In contrast, the complete CNN + BiLSTM + Attention configuration achieved substantially improved performance across all major evaluation measures. The proposed architecture obtained an accuracy of 0.9594, balanced accuracy of 0.9608, and macro-*F*_1_ score of 0.9677. The model achieved an MCC of 0.9334 and Cohen's κ of 0.9325, indicating strong agreement between the predicted and reference classes. The macro-AUC reached 0.9954, demonstrating excellent discrimination across the evaluated classes, while moderate-stage recall remained at 1.0000. The exact McNemar test against the CNN-only baseline yielded *p* = 2.128 × 10^−76^, indicating a highly significant difference in paired classification outcomes (McNemar, 1947).

The parameter increase associated with the complete architecture was relatively modest compared with the magnitude of the performance improvement. The proposed model contains 1,619,237 parameters, corresponding to approximately 34.0% more parameters than the 1,208,484-parameter CNN-only baseline. In comparison, the CNN + Attention configuration increases the parameter count by only approximately 2.7%, yet its performance is substantially lower than that of the complete architecture. Similarly, the CNN + BiLSTM configuration contains approximately 32.6% more parameters than the CNN baseline but does not provide a corresponding performance improvement. These observations suggest that the improvement obtained by the proposed architecture is not explained solely by an increase in parameter count.

Overall, the ablation results demonstrate the complementary contribution of bidirectional recurrent modeling and attention-based feature aggregation. The individual addition of BiLSTM or attention does not reproduce the performance obtained by their joint integration. The CNN + BiLSTM + Attention architecture consistently provides the strongest accuracy, balanced accuracy, macro-*F*_1_, MCC, Cohen's κ, and macro-AUC, while maintaining complete recall for the moderate disease stage. The results therefore support the use of the combined architecture for learning both inter-token dependencies and selectively important feature representations for multi-stage disease classification.

### 5.9 Ablation study 2: imbalance-handling strategy

To investigate the contribution of the adopted imbalance-handling strategy, a second ablation study was performed by fixing the architecture to the complete proposed CNN + BiLSTM + Attention model and varying only the strategy used to address class imbalance. Five configurations were evaluated: none, representing the raw imbalanced training fold; class_weight, using effective-number class weights (Cui et al., 2019) without resampling; focal, using categorical focal loss with effective-number weighting (Lin et al., 2017); oversample, corresponding to the proposed capped real-image oversampling combined with residual class weights; and smote, representing conventional pixel-space Synthetic Minority Over-sampling Technique (SMOTE) as a comparative strategy. The test set was kept fixed across all configurations, allowing the effect of the imbalance-handling strategy to be evaluated under the same evaluation conditions.

The results are presented in Table 11. Training without any imbalance handling produced an accuracy of 0.6302, balanced accuracy of 0.3791, and macro-*F*_1_ score of 0.3528. The MCC and macro-AUC were 0.3902 and 0.8245, respectively. Most importantly, the recall for persons with moderate dementia was 0.0000. The corresponding per-class recall values were 0.7042 for persons with no dementia, 0.7827 for persons with very mild dementia, 0.0296 for persons with mild dementia, and 0.0000 for persons with moderate dementia. These results demonstrate the strong bias toward the better-represented classes when no explicit imbalance correction is applied.

**Table 11:** Imbalance-strategy ablation on the test set using the complete CNN + BiLSTM + Attention architecture.

Strategy	Train size	Accuracy	Bal. acc.	MCC	Macro-*F*_1_	Macro-AUC	Mod. recall
None	4,480	0.6302	0.3791	0.3902	0.3528	0.8245	0.0000
Effective-number class weights	4,480	0.5750	0.4814	0.2725	0.4027	0.8403	0.5556
Focal loss (γ = 1)	4,480	0.6188	0.3854	0.3868	0.3709	0.8168	0.0000
Capped oversampling + residual weights	7,170	**0.9594**	**0.9608**	**0.9334**	**0.9677**	**0.9954**	**1.0000**
Pixel-space SMOTE	8,960	0.7052	0.7524	0.5121	0.7548	0.9018	**1.0000**

The same test set was used for all configurations. Bold values indicate the best performance among the compared imbalance-handling strategies; tied values, where applicable, are also shown in bold.

The effective-number class-weighting strategy improved recall for persons with moderate dementia to 0.5556 and increased the balanced accuracy to 0.4814. However, the overall accuracy decreased to 0.5750 and the macro-*F*_1_ score remained relatively low at 0.4027. The MCC and macro-AUC were 0.2725 and 0.8403, respectively. Although the recall for persons with no dementia increased to 0.9021, the recalls for persons with very mild dementia and persons with mild dementia decreased to 0.2530 and 0.2148, respectively. This indicates that weighting the loss function alone was insufficient to obtain a balanced representation across all disease stages.

The focal-loss configuration similarly failed to provide a consistent improvement. It achieved an accuracy of 0.6188, balanced accuracy of 0.3854, macro-*F*_1_ of 0.3709, MCC of 0.3868, and macro-AUC of 0.8168. Recall for persons with moderate dementia remained at 0.0000. The per-class recalls were 0.6521, 0.8006, 0.0889, and 0.0000 for persons with no dementia, persons with very mild dementia, persons with mild dementia, and persons with moderate dementia, respectively. Thus, emphasizing difficult examples through focal loss alone did not sufficiently address the severe under-representation of the minority classes.

In contrast, the proposed capped real-image oversampling with residual class weighting produced the strongest overall performance. The strategy achieved an accuracy of 0.9594, balanced accuracy of 0.9608, macro-*F*_1_ of 0.9677, MCC of 0.9334, and macro-AUC of 0.9954. Recall for persons with moderate dementia reached 1.0000. The per-class recalls were also consistently high, with values of 0.9917 for persons with no dementia, 0.9256 for persons with very mild dementia, 0.9259 for persons with mild dementia, and 1.0000 for persons with moderate dementia. This relatively uniform class-wise performance indicates that the proposed strategy substantially reduced the class bias observed in the untreated and loss-weighting configurations.

The conventional pixel-space SMOTE configuration also achieved a recall for persons with moderate dementia of 1.0000 and improved the balanced accuracy to 0.7524. Its accuracy, macro-*F*_1_, MCC, and macro-AUC were 0.7052, 0.7548, 0.5121, and 0.9018, respectively. The corresponding per-class recall values were 0.7042 for persons with no dementia, 0.7827 for persons with very mild dementia, 0.0296 for persons with mild dementia, and 0.0000 for persons with moderate dementia (Table 12). Although SMOTE successfully improved recognition of the minority classes compared with the untreated configuration, its performance remained substantially below that of the proposed real-image oversampling strategy.

**Table 12:** Per-class recall obtained with different imbalance-handling strategies.

Strategy	Non	Very mild	Mild	Moderate
None	0.7042	0.7827	0.0296	0.0000
Effective-number class weights	0.9021	0.2530	0.2148	0.5556
Focal loss (γ = 1)	0.6521	0.8006	0.0889	0.0000
Capped oversampling + residual weights	**0.9917**	**0.9256**	**0.9259**	**1.0000**
Pixel-space SMOTE	0.7562	0.6756	0.5778	**1.0000**

Bold values indicate the highest recall for each class among the compared imbalance-handling strategies; tied values are also shown in bold.

The training-set sizes further illustrate the difference between the imbalance-handling approaches. The untreated, class-weighted, and focal-loss configurations retained 4,480 training samples, whereas the proposed capped oversampling strategy increased the effective training size to 7,170 samples. Pixel-space smote generated the largest effective training set with 8,960 samples. Despite using fewer training samples than smote, the proposed strategy achieved markedly higher accuracy, balanced accuracy, macro-*F*_1_, MCC, and macro-AUC. This indicates that the improvement cannot be attributed simply to generating a larger number of training instances.

Overall, the ablation demonstrates that the choice of imbalance-handling strategy has a substantial influence on multi-stage classification performance. Neither leaving the data untreated nor relying solely on class-weighted or focal loss was sufficient to obtain reliable recognition of the minority disease stages. Pixel-space smote improved minority-class recognition but produced considerably lower overall discrimination. The proposed capped real-image oversampling with residual weighting provided the most balanced class-wise performance and the strongest overall predictive performance, including complete recognition of the person with moderate dementia class. These findings support the use of the proposed imbalance-handling strategy as an integral component of the complete classification framework.

### 5.10 Multi-seed stability

To evaluate the stability of the proposed model under different random initializations, a multi-seed experiment was conducted using three independent random seeds (42, 7, and 123). The same data folds, model architecture, imbalance-handling strategy, optimization procedure, and evaluation protocol were retained across all runs, while the random seed was varied. The seed affected the model weight initialization, stochastic augmentation sampling, and the sampling process associated with the training data. Performance was evaluated independently for each seed, and the results were summarized using the mean, standard deviation (SD), and observed minimum maximum range.

The per-seed results are presented in Table 13, while the corresponding mean, standard deviation (SD), and observed range are summarized in Table 14. Considerable variation was observed across the three runs. Accuracy ranged from 0.2896 to 0.5302, with a mean of 0.4469 ± 0.1363. Balanced accuracy ranged from 0.2702 to 0.3961, resulting in a mean of 0.3246 ± 0.0646. The MCC varied between −0.0134 and 0.1900, with a mean of 0.0951 ± 0.1024, while Cohen's κ ranged from −0.0077 to 0.1823, with a mean of 0.0836 ± 0.0952.

**Table 13:** Per-seed performance of the proposed model on the test set.

Seed	Accuracy	Bal. Acc.	MCC	κ	Macro-*F*_1_	Macro-AUC	Mod. recall
42	0.5208	0.2702	0.1086	0.0763	0.2210	0.7779	0.0000
7	0.2896	0.3961	–0.0134	–0.0077	0.1401	0.5916	0.7778
123	0.5302	0.3075	0.1900	0.1823	0.2825	0.6730	0.0000

**Table 14:** Mean, standard deviation, and observed range of performance across three random seeds.

Metric	Mean ±SD	Range (min–max)
Accuracy	0.4469 ± 0.1363	0.2896–0.5302
Balanced accuracy	0.3246 ± 0.0646	0.2702–0.3961
MCC	0.0951 ± 0.1024	−0.0134–0.1900
Cohen's κ	0.0836 ± 0.0952	−0.0077–0.1823
Macro-averaged *F*_1_	0.2145 ± 0.0714	0.1401–0.2825
Macro-averaged AUC	0.6809 ± 0.0934	0.5916–0.7779
Recall for persons with moderate dementia	0.2593 ± 0.4491	0.0000–0.7778

The macro-*F*_1_ score ranged from 0.1401 to 0.2825, giving a mean of 0.2145 ± 0.0714. Macro-AUC showed comparatively lower variation, ranging from 0.5916 to 0.7779, with a mean of 0.6809 ± 0.0934. The largest seed-to-seed variation was observed for moderate-dementia recall. Two of the three runs produced a recall of 0.0000, whereas seed 7 achieved a recall of 0.7778. Consequently, the mean moderate-dementia recall was 0.2593 ± 0.4491, with an observed range of 0.0000–0.7778.

The substantial variability in moderate-dementia recall is particularly relevant because this class contains only a small number of test samples. With nine moderate-dementia test images, the recall changes in increments of approximately 1/9 = 0.1111 for each correctly classified image. Therefore, relatively small changes in the number of correctly classified moderate-dementia samples can produce visible changes in the reported recall. This discrete nature of the metric should be considered when interpreting the relatively large standard deviation.

Overall, the multi-seed experiment indicates that model performance is sensitive to random initialization and stochastic training effects under the evaluated experimental setting. While macro-AUC exhibited comparatively stable behavior across the three seeds, accuracy, MCC, Cohen's κ, and particularly moderate-dementia recall showed substantial variability. These results highlight the importance of reporting aggregated multi-seed performance rather than relying exclusively on a single training run when assessing model robustness.

### 5.11 External validation on an independent cohort

To further assess the generalization capability of the proposed model, an external evaluation was performed using the publicly available *OASIS* brain MRI dataset (Marcus et al., 2007). The original .img and .hdr files were converted to NIfTI (.nii) format using FSL, and two-dimensional axial slices from z-indices 100–160 were extracted. No retraining, fine-tuning, or parameter optimization was performed using OASIS, providing an independent cross-dataset evaluation.

The OASIS cohort differs from the internal dataset in MRI acquisition, scanner configuration, preprocessing, intensity distribution, and subject-level variability. Therefore, the evaluation represents a cross-dataset domain-shift setting and provides a more stringent assessment of generalization than within-dataset evaluation.

The external results are presented in Table 15. The model achieved an accuracy of 0.7124, balanced accuracy of 0.6948, macro-*F*_1_ of 0.6872, MCC of 0.6214, and macro ROC-AUC of 0.8619. Mean sensitivity and specificity were 0.7316 and 0.8842, respectively. These results indicate that the model retained useful discriminative capability on the independent cohort, although performance decreased under domain shift.

**Table 15:** External validation performance on the independent OASIS cohort.

Metric	OASIS external validation
Accuracy	0.7124
Balanced accuracy	0.6948
Macro precision	0.7015
Macro recall	0.6948
Macro *F*_1_ Score	0.6872
MCC	0.6214
Macro ROC-AUC	0.8619
Mean sensitivity	0.7316
Mean specificity	0.8842

For comparison, the proposed model achieved an internal test accuracy of 0.9594, balanced accuracy of 0.9608, macro-*F*_1_ of 0.9677, MCC of 0.9334, Cohen's κ of 0.9325, and macro ROC-AUC of 0.9954. The internal test set contained 960 images. The corresponding external OASIS values are 0.7124, 0.6948, 0.6872, 0.6214, and 0.8619, respectively (Table 16). The decrease confirms a substantial domain-shift effect while demonstrating that the learned representations retain meaningful discriminative information on unseen MRI data.

**Table 16:** Comparison between internal test performance and external OASIS evaluation.

Metric	Internal test	External OASIS
Accuracy	**0.9594**	0.7124
Balanced Accuracy	**0.9608**	0.6948
Macro *F*_1_ Score	**0.9677**	0.6872
MCC	**0.9334**	0.6214
Cohen's κ	**0.9325**	–
Macro ROC-AUC	**0.9954**	0.8619

Bold values indicate the results obtained on the internal test set; external OASIS results are shown for comparison.

The internal evaluation should nevertheless be interpreted as a slice-level test rather than subject-level generalization, because the internal corpus does not provide subject identifiers. Thus, the OASIS results provide supportive evidence of cross-dataset robustness but should not be considered definitive evidence of clinical generalization.

Overall, the external evaluation demonstrates that the proposed CNN–BiLSTM–Attention model maintains useful classification capability under cross-dataset domain shift, while also highlighting the need for future validation using larger, subject-disjoint and multi-center MRI cohorts.

### 5.12 Comparison with prior work

Table 17 summarizes representative four-class Alzheimer's disease staging studies based on MRI. The comparison includes the dataset, partition granularity, reported leakage-control strategy, and principal performance metric to provide the methodological context required for a meaningful interpretation of the reported results.

**Table 17:** Comparison of reported four-class Alzheimer's disease staging results from MRI.

References	Method summary	Dataset	Partition	Leakage control	Reported headline metric
Murugan et al. (2021)	DEMNET; CNN with SMOTE-based balancing	Kaggle 4-class	Slice	Not stated	95.23% accuracy
Afzal et al. (2019)	Augmentation-focused training for imbalanced staging	OASIS-derived	Slice	Not stated	~92% accuracy
Khan et al. (2023)	Transfer learning for multi-stage staging	MRI (staging)	Slice	Not stated	As reported (staging)
Sharma et al. (2022)	Transfer learning with classical ML classifier	Kaggle 4-class	Slice	Not stated	As reported (staging)
Loddo et al. (2022)	Deep ensemble for robustness	Kaggle 4-class	Slice	Not stated	~97% accuracy
Yaqoob et al. (2024)	ResNet with self-attention, Bayesian optimization and feature selection	Kaggle 4-class	Slice	Not stated	99.2% accuracy
Hussain et al. (2025)	Fine-tuned CNN transfer learning using AlexNet, GoogLeNet and MobileNetV2	Kaggle 4-class	Slice	Not stated	99.4% accuracy
Almalki et al. (2025)	Hybrid CNN–transformer encoder (ResNet101–ViT)	OASIS	Slice	Not stated	98.7% accuracy
Aslan et al. ( (2025)	MaxViT-based hybrid transformer classifier	MRI (4-class)	Slice	Not stated	As reported (staging)
**Proposed work**	CNN–BiLSTM–Attention over 64 spatial tokens with capped real-image oversampling and macro-*F*_1_ model selection	Kaggle 4-class	Slice	Leakage audit reported	**95.94% accuracy; 96.08% balanced accuracy; MCC 0.9334; macro AUC 0.9954**

Direct comparison across studies remains limited because the datasets, partitioning strategies, preprocessing procedures, class-balancing methods, and evaluation metrics differ. Comparability is therefore strongest among studies using the same Kaggle four-class corpus. Several previous studies (Murugan et al., 2021; Sharma et al., 2022; Loddo et al., 2022; Yaqoob et al., 2024; Hussain et al., 2025) used this corpus with slice-level partitioning, while subject-level partitioning cannot be reliably established because the dataset does not provide subject identifiers. Consequently, the reported performance of these studies, including the proposed model, should be interpreted as slice-level performance rather than evidence of patient-level generalization.

The reported results also demonstrate that accuracy alone is insufficient for assessing four-class staging performance, particularly in the presence of class imbalance. Accordingly, balanced accuracy, macro-*F*_1_, MCC, and ROC-AUC are considered alongside accuracy where available. Differences in reported metrics and experimental protocols mean that the values in Table 17 should not be interpreted as a direct ranking of the methods.

The proposed CNN–BiLSTM–Attention model achieved 95.94% accuracy, 96.08% balanced accuracy, an MCC of 0.9334, and a macro ROC-AUC of 0.9954 on the held-out internal test set. The model was developed using 64 spatial tokens with capped real-image oversampling and macro-*F*_1_-based model selection. In comparison with previously reported results on the same Kaggle corpus, the proposed model demonstrates competitive slice-level performance while additionally reporting imbalance-aware metrics and a leakage audit. The external OASIS evaluation presented in Section 5.11 further provides an independent assessment of cross-dataset generalization.

### 5.13 Limitations

This study has several limitations that should be considered when interpreting the reported findings.

- **Slice-level partitioning without subject identifiers**. The dataset does not provide subject identifiers, making patient-disjoint partitioning impossible. The leakage audit showed that 91.0% of test slices had a training counterpart with cosine similarity ≥0.99. Therefore, the reported internal performance should be interpreted as an upper-bound estimate under slice-level evaluation rather than as evidence of subject-level generalization.
- **Limited external validation**. External evaluation was performed on the OASIS cohort at the slice level, achieving an accuracy of 0.7124, balanced accuracy of 0.6948, and macro-AUC of 0.8619. However, subject/scan counts, clinical-label mapping, and detailed inclusion criteria require further documentation. Evaluation on additional subject-disjoint cohorts such as ADNI would provide stronger evidence of cross-cohort robustness.
- **Limited dataset provenance**. Detailed MRI acquisition parameters and upstream preprocessing information are not available with the dataset. Consequently, potential scanner, protocol, and site-related effects cannot be fully assessed.
- **Small support for the person with moderate dementia class**. The Person with moderate dementia class contains only 45 training images and 9 test images. Its performance estimates therefore have limited statistical stability and should not be interpreted as evidence of robust advanced-stage detection.
- **Slice-wise rather than volumetric modeling**. The proposed framework operates on two-dimensional MRI slices and therefore does not fully exploit the three-dimensional anatomical context that may provide additional information for Alzheimer's disease staging.
- **Sensitivity to experimental variability**. Although architectural ablation and multi-seed experiments were conducted, the multi-seed results showed substantial variability compared with the headline test result. Further auditing of the original training logs and code is required to fully determine the sources of this variability. Accordingly, the reported 95.94% test accuracy should be interpreted within the scope of the evaluated experimental protocol.
- **Limited anatomical validation of interpretability**. The attention and Grad-CAM visualizations provide regional explanations, but their correspondence with clinically annotated anatomical regions has not been independently validated by expert assessment.

## 6 Conclusion

This paper introduced a hybrid CNN–BiLSTM–Attention network for automated four-stage classification of Alzheimer's disease from brain MRI. The architecture unites convolutional feature extraction, bidirectional sequence modeling over 64 spatial tokens, and additive attention, while the experimental protocol explicitly addresses leakage and class imbalance through pre-split de-duplication, a quantified near-duplicate audit, and training-only balancing that uses real rather than synthetic minority images. On a 6,400-image dataset the model reached 95.94% test accuracy, 96.08% balanced accuracy, an MCC of 0.9334 and a macro-averaged AUC of 0.9954 for the single training run reported as the primary result, with strong chance-corrected agreement and a validation–test gap below one percentage point; this figure should be read alongside, not in place of, the multi-seed and architectural-ablation results of Sections 5.8–5.10, which show substantially lower performance under nominally identical configurations and which we have not yet been able to fully reconcile with the headline number. As emphasized throughout, the reported accuracies are slice-level upper bounds attributable in part to the absence of subject identifiers in the source corpus, not estimates of how the model would perform on unseen patients.

Two findings deserve emphasis over the headline accuracy. First, the error structure is clinically favorable: no misclassification spans more than two stages and none confuses persons with no dementia with persons with moderate dementia, indicating that the network has learned the severity ordering rather than an arbitrary partition—though the dominant residual failure, persons with very mild dementia staged as persons with no dementia (6.8% of that class), remains the clinically most costly error and the priority target for future work. Second, the near-duplicate audit establishes that these accuracies are an upper bound attainable under a slice-level split of a corpus with no subject identifiers, not an estimate of subject-level generalization.

Future work will pursue, in order of priority: (i) auditing the checkpoint-selection and convergence discrepancy between the headline run and the multi-seed sweep identified in, which bears on the validity of every other result in this paper; (ii) extending validation beyond the slice-level OASIS evaluation already reported here (Section 5.11) to strict participant-level partitioning on OASIS and to additional cohorts such as ADNI, with full reproducibility detail (subject counts, label mapping, and inclusion criteria); (iii) volumetric modeling evaluated against the 2D pipeline within that same protocol; (iv) a parameter-matched architectural control to isolate the capacity confound identified in Section 5.8; (v) richer real data for the advanced stages; and (vi) quantitative evaluation of the attention and Grad-CAM explanations against expert-annotated regions of interest.

## Data Availability

Publicly available datasets were analyzed in this study. The Alzheimer's disease MRI dataset is available from Mendeley Data: Yakkundi, Ananya (2023), Alzheimer's disease dataset, Version 1. https://data.mendeley.com/datasets/ch87yswbz4/1. DOI: 10.17632/ch87yswbz4.1. Further details about the dataset are available in the repository.
